# Ionogels Based on a Single Ionic Liquid for Electronic Nose Application

**DOI:** 10.3390/chemosensors9080201

**Published:** 2021-07-30

**Authors:** Wellington B. Gonçalves, Evelyn P. Cervantes, Ana C. C. S. Pádua, Gonçalo Santos, Susana I. C. J. Palma, Rosamaria W. C. Li, Ana C. A. Roque, Jonas Gruber

**Affiliations:** 1 Departamento de Química Fundamental, Instituto de Química, Universidade de São Paulo, Av. Prof. Lineu Prestes, 748, São Paulo 05508-000, SP, Brazil; 2 Departamento de Ciência da Computação, Instituto de Matemática e Estatística, Universidade de São Paulo, Rua do Matão, 1010, São Paulo 05508-090, SP, Brazil; 3 UCIBIO, Departamento de Química, Faculdade de Ciências e Tecnologia, Universidade Nova de Lisboa, 2829-516 Caparica, Portugal; 4 Centro Universitário UniBTA, Rua Afonso Sardinha, 201, São Paulo 05076-000, SP, Brazil

**Keywords:** composite, electronic nose, gas sensor, ionogel, volatile organic compound, ionic liquid

## Abstract

Ionogel are versatile materials, as they present the electrical properties of ionic liquids and also dimensional stability, since they are trapped in a solid matrix, allowing application in electronic devices such as gas sensors and electronic noses. In this work, ionogels were designed to act as a sensitive layer for the detection of volatiles in a custom-made electronic nose. Ionogels composed of gelatin and a single imidazolium ionic liquid were doped with bare and functionalized iron oxide nanoparticles, producing ionogels with adjustable target selectivity. After exposing an array of four ionogels to 12 distinct volatile organic compounds, the collected signals were analyzed by principal component analysis (PCA) and by several supervised classification methods, in order to assess the ability of the electronic nose to distinguish different volatiles, which showed accuracy above 98%.

## 1 Introduction

Ionic liquids (ILs) are compounds widely studied in several areas of knowledge due to their low vapor pressure, high thermal and chemical stability, non-flammability, and high electrical conductivity [1]. These properties allow the use of ILs as solvents [2], catalysts [3], and electrolytes [4], among others [5]. However, the use of ILs in electronic devices, such as electrochemical sensors, is challenging due to their low-dimensional stability [6]. One way to overcome this problem is to immobilize the ILs in a solid three-dimensional matrix, which can be organic, inorganic, or hybrid (organic–inorganic), forming ionogels that preserve the main properties of the ILs but give them a quasi-solid behavior [6,7].

Ionogels are widely used in applications such as drug carriers [8], lithium batteries [9], fuel cells [10], solar cells [11], sensors [12,13], catalysts [14,15], and as active layers in chemiresistive gas sensors and electronic noses (e-noses) [16].

An e-nose is a device made of an array of gas sensors connected to a pattern recognition system capable of recognizing and analyzing volatiles of pure substances and even of complex mixtures [17]. These mixtures of volatile organic compounds (VOCs) are formed in many biological and chemical processes enabling the application of e-noses in many areas such as agriculture [18], food monitoring [19–21], environmental science [21], forestry [22], military [23], industry [24], bacterial detection [25], and disease diagnosis [26], especially through metabolomics [27].

Composite materials present improved characteristics compared to the original polymers, and also, new functionalities can be introduced. This makes them suitable for a wide range of applications, for instance, in aeronautic [28] and automotive [29] industries, and in gas sensing [30]. Some examples of their application in e-noses follow: Zheng et al. [31] developed an e-nose composed by functionalized carbon nanotubes added to three different polymeric matrixes. The sensors were employed in a wearable e-nose to identify human skin odors that can serve as “fingerprint” for individual identification. Swe et al. [32] applied nanocomposites in an e-nose for monitoring beef freshness level. The sensors based on polymeric matrix and single wall carbon nanotubes were capable of distinguishing the concentration of different volatile compounds exhaled from beef before and after spoilage.

Carvalho et al. [33] first described the use of ionogel in e-noses. Four ionogels, employing distinct ILs trapped in a single biopolymer (bovine gelatin), were used as active layers in gas sensors, forming an e-nose capable of distinguishing eight VOCs with fast response and good reproducibility. Netto et al. [16] reported an e-nose formed by an array of three gas sensors made of a single ionic liquid (EMIMDCA) and three different biopolymers (bovine gelatin, agar, and sodium alginate) and tested with five VOCs giving a hit rate > 96%. Gelatin ionogels have also shown the ability to encapsulate additional probes, such as liquid crystal droplets, to increase selectivity and sensitivity in gas sensing [34]. Some more application examples of this type of sensors are the quantification of ethanol in ethanol–gasoline fuel mixtures [34], *Tilapia* fish freshness monitoring [35], and a benzene and formaldehyde sensor [36].

In this work, we designed and developed a new composite material formed by a single gelatin–ionogel doped with iron oxide (Fe_3_O_4_) particles for application as sensitive layers in gas sensors for e-noses. First, we studied the influence of different concentrations of bare Fe_3_O_4_ particles on the electrical response of the material. Then, we kept the concentration constant and functionalized the particles with oleic acid, lauric acid, and anthracene carboxylic acid, aiming to increase the interaction between the ionogel and the VOCs. These ionogels were used as active layers in gas sensors for e-noses that were exposed to model VOCs, and the collected signals were examined by principal component analysis (PCA) and validation methods. It is worth mentioning that varying only the concentration of one of the components or its functionalization can greatly simplify the process of manufacturing gas sensors and e-noses.

## 2 Materials and Methods

### 2.1 Materials

**Figure 1:**
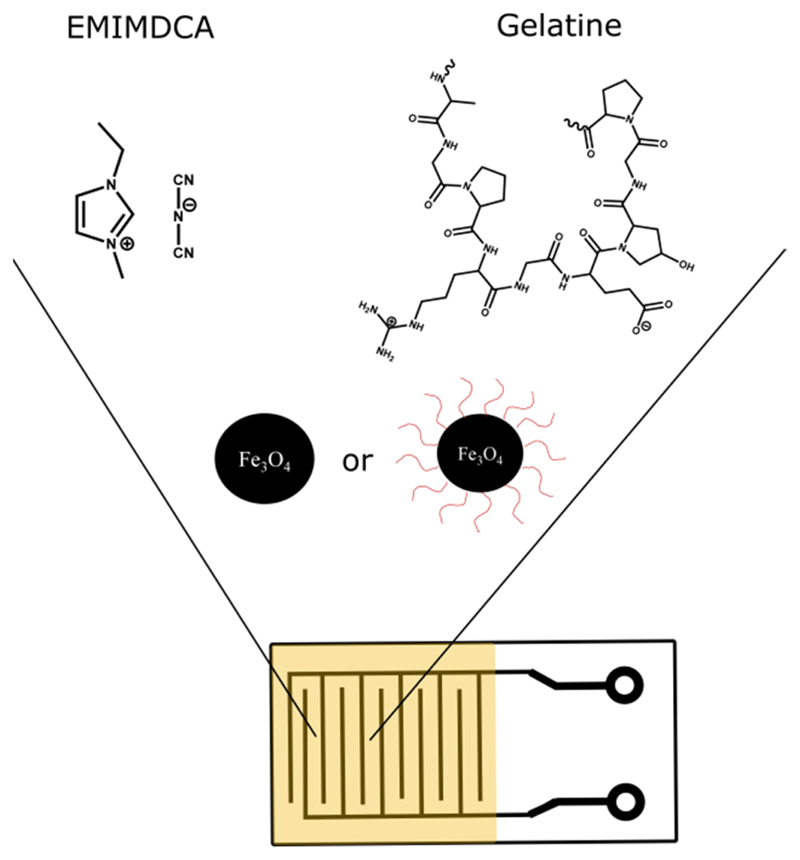
Interdigitated electrode used in the preparation of the gas sensors.

1-Ethyl-3-methylimidazolium dicyanamide (EMIMDCA, >98%), type B bovine skin gelatin, lauric acid (LA, ≥99%), and anthracene carboxylic acid (AA, ≥99%) were purchased from Sigma Aldrich. Iron(II) chloride tetrahydrate (FeCl_2_.4H_2_O, ≥99%) was obtained from Vetec, iron(III) chloride hexahydrate (FeCl_3_.6H_2_O, >99%) was supplied by Acros Organic. Oleic acid (OA, ≥99%) was purchased from Carl Roth. Ammonium hydroxide solution and all organic solvents were purchased from Labsynth Produtos para Laboratórios Ltd.a and used as received. The interdigitated electrodes were manufactured by Micropress S. A. with 0.6 cm^2^ of interdigitated area and 200 μm spacings between the copper digits (Figure 1).

### 2.2 Preparation and Characterization of Iron Oxide Particles

Iron oxide particles were produced according to established methods [37]. In summary, 6.0 g of FeCl_3_.6H_2_O were dissolved in 100 mL of distilled water, and 2.7 g of FeCl_2_.4H_2_O were dissolved in 11 mL of distilled water. Both solutions were mixed and mechanically stirred under nitrogen atmosphere until homogenization. Then, 19 mL of ammonium hydroxide solution (25%) were quickly added to the mixture, which was allowed to react for 5 min. Stirring was discontinued, the mechanical stirrer was removed and the Fe_3_O_4_ particles were separated by magnetic decantation. The bare particles were washed with water several times and dispersed in water with a final concentration of 78 mg/mL.

To generate functionalized particles, 1.4 mmol aliquots of the bare particles dispersed in water were mixed with 4.1 mmol of OA in 8.0 mL n-hexane, with 4.1 mmol of LA in 8.0 mL of n-hexane or with 4.1 mmol AA in 8.0 mL of DMSO. The mixtures were mechanically stirred for 15 h under nitrogen atmosphere. The organic phase became black, while the aqueous phase became colorless, which is consistent with the migration of the hydrophobic functionalized particles from the aqueous to the organic layer. After separation of the organic phases containing OA and LA particles, the suspensions were stored. The AA particles were decanted magnetically, washed with distilled water, and dispersed in acetonitrile.

A Fourier transform infrared spectrometer (FTIR), PerkinElmer model Frontier FR-IR, was used to characterize the surface of the bare particles and AA, LA, and OA coated particles. The particles were ground and mixed with KBr to prepare the sample disks.

The hydrodynamic diameter of the bare particles was determined using a Malvern dynamic light scattering particle size analyzer (Zetasizer Nano Zs90) operating at 25 °C.

### 2.3 Ionogel Preparation

Control ionogels (without particles) were prepared as previously described [27]. In summary (Table 1, entries 1–4), 30 mg of bovine skin gelatin were added to 75 μL of EMIMDCA, followed beys 40 μL of distilled water. The mixture was kept in an ultrasonic bath for 15 min. For the preparation of ionogels doped with bare particles, three different concentrations of a suspension of the particles in distilled water (25 mg/mL, 50 mg/mL and 75 mg/mL) were added, generating three different composites. To generate ionogels doped with functionalized particles (Table 1, entries 5–7), 40 μL aliquots of solutions (225 mg/mL) of the functionalized particles were added to the gelatin. The solvent was evaporated, and the doped gelatin was mixed with EMIMDCA as described above.

### 2.4 Sensor Preparation and Application in e-Nose

The sensors were divided into two sets: the first (A) formed by the pure ionngel and by doped i onegel with the three different bare particle concentrations (Table 1, entries 1–4); the second (B) formed by the doped ionogel (25 mg/mL) with bare and functionalized particles (Table 1, entries 2 and 5–7). Then, 40 μL of the ionogel were applied on the active part of the interdigitated electrode (Figure 1) and spin coated at 10,000 rpm for 5 s, forming a thin film. The sensors produced were dried in a desiccator under vacuum for 15 h.

**Figure 2:**
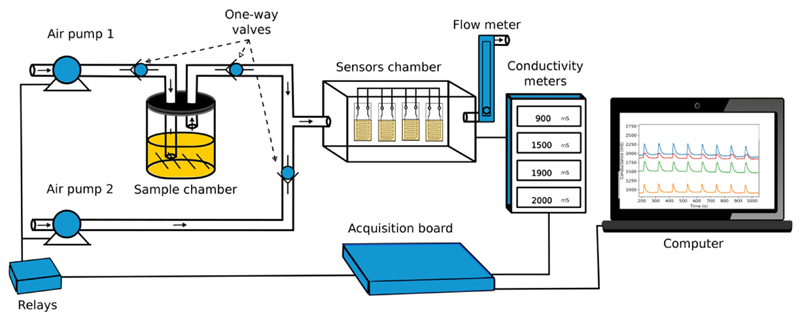
Schematic representation ol the electronic nose assembled.

The sensors were tested in an e-nose system with dynamic sampling built in our laboratory and shown, as a schematic representation, in Figure 2. A complete measurement cycle consisted of an exposure phase (pump 1 on, pump 2 off), where the sample headspace was transported to the sensors’ chamber, and a recovery phase (pump 2 on, pump 1 off), where ambient air passed directly through the sensors, removing volatiles and regenerating the sensors. Thrne neon-return valves were used to ensure that air flow was always in the sensors’ direction. Each analyte was kept in the sample compartment thermostated at 30 °C throughout the experiment, and the airflow was maintained at 0.5 L/min. For all analytes, the exposure (5 s)/recovery (100 s) cycle was repeated 10 times, and the first two cycles were discarded to avoid the influence of volatiles from previous samples that could be still present in the system. The conductance was registered by a conductivity meter [38], operating with 80 mV peak-to-peak 2 kHz triangle wave ac voltage. and connected through a 10-bit analog-to-digital converter to a personal computer. The sensors did not require eny cleaning or preparation between samples. The temperature and humidity of the laboratory were maintained at 23 °C and 60%, respectively, during all experiments.

### 2.5 Data Treatment

**Figure 3:**
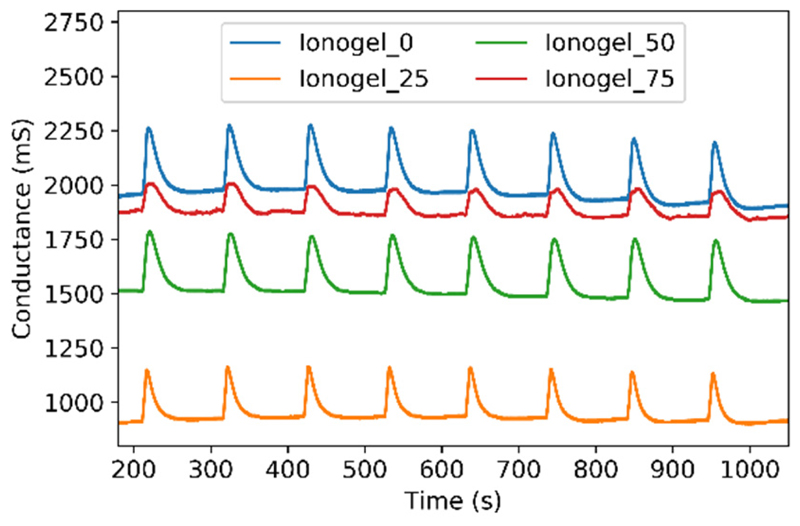
Response of set A sensors to eight exposure (5 s)/recovery (100 s) cycles to ethanol vapors, doped with different concentrations of Fe_3_O_4_: (blue) pure ionogels, (orange) 25 mg/mL, (green) 50 mg/mL and (red) 75 mg/mL.

The VOCs used as model compounds were organic solvents with different functional groups, polarities, and boiling points: ethanol, acetone, chloroform, ethyl acetate, toluene, and hexane. Both sets of sensors (A and B) were tested with these solvents. The interaction of the solvents with the sensors led to changes in their electrical conductance. A typical conductance response of the sensors (set A) during the last 8 exposure (5 s)/recovery (100 s) cycles to ethanol vapors is shown in Figure 3.

The data were standardized calculating the relative responses (Ra) of all cycles of the tested solvents according to Equation (1), where G2 is the conductance at the end of the exposure period to the solvent vapor and G1 is the initial conductance before this exposure period.

(1) Ra=(G2−G1)/G1

These calculated Ra values for the last eight cycles of the sensors were used as input data in principal component analysis (PCA), generating clusters that can be associated with the analyzed volatiles. Then, supervised methods were applied to verify the prediction capability of the e-nose using the K-fold cross-validation method, with k value equal to 10. In this method, the data were divided into k groups, where k-1 groups were used to train the classifier and one group was used to test the accuracy. This process was repeated k times.

## 3 Results and Discussion

### 3.1 Characterization of Fe_3_O_4_

The bare particles were synthesized, using the co-precipitation method previously described by Palma et al. [37]. The average particle diameter (168 nm) and the polydispersity index (0.351) were obtained by means of dynamic light scattering (DLS).

**Figure 4:**
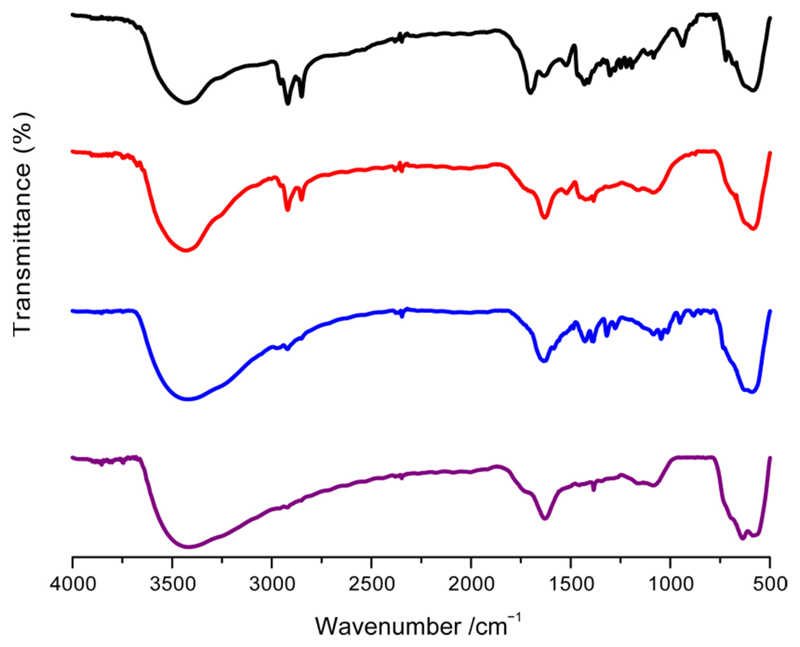
FTIR spectra of bare Fe_3_O_4_ particles (magenta) and of functionalized Fe_3_O_4_ particles with AA (blue), OA (red), and LA (black).

Functionalizations were confirmed using infrared spectroscopy (FTIR KBr) (Figure 4). The absorptions of bare particles can be seen at 580 cm^−1^, corresponding to the Fe-O bond of the Fe_3_O_4_ crystalline structure, and at 1630 and 3405 cm^−1^, which correspond to hydroxyl groups present on their surface due to the synthetic method used to prepare them [39]. Comparatively, the spectrum of particles functionalized with AA shows bands at 2921 cm^−1^ (aromatic C-H stretch), at 1428 and 1385 cm^−1^ bending of the C-O-H group of the carboxylic acid), at 1318 and 1276 cm^−1^ (C-O stretch), and at 951 cm^−1^ (COH out of plane bending) [40,41]. The particles functionalized with LA and OA present similar spectra, with bands between 2925 and 2840 cm^−1^ (asymmetric and symmetric stretches of the C-H bond of CH_2_), and at 1522 and 1464 cm^−1^ (asymmetric and symmetric stretches of -COO bonds of the carboxyl group) [39]. In summary, characteristic aromatic C-H stretch bands are only present in the spectrum of AA-functionalized particles, as they are the only ones with aromatic rings (anthracene). On the other hand, aliphatic C-H stretch bands appear in the spectra of particles bound to LA and OA due to their long aliphatic chains, which do not exist in the other particles. Similar conclusions can be made when analyzing the other characteristic bands mentioned above.

### 3.2 The Sensors

The ionogel composite is formed by an organized polypeptide structure (gelatin), IL, water, and Fe_3_O_4_ particles. The IL is an important component responsible for electrical conductivity and solvation capacity. The fast response (5 s) and recovery (100 s) of the sensor suggests a fairly good solvation of the VOCs in the IL but with weak interactions, so that adsorption/desorption process can occur promptly [33,42], The sensing mechanism can be attributed to changes in the IL viscosity. As the IL absorbs and dissolves a VOC, the latter inhibits cation–anion interactions, resulting in decreased IL viscosity, improving ionic mobility and consequently increasing conductance [43]. In addition, Fe_3_O_4_ affects the cation–anion interaction, leading to a change in ionic mobility and different response patterns depending on the Fe_3_O_4_ concentration.

Although the fast response of the ionogel indicates good solvation and adsorption of the analytes, there are some VOCs with greater affinity to the ionogel than others. This is because VOCs with medium to high dielectric constants are more miscible with IL than those with low dielectric constants [44,45], which generally show signals with much lower amplitudes. To overcome this limitation, we proposed to functionalize the Fe_3_O_4_ particles with molecules that have chemical structures similar to those of VOCs with a low dielectric constant, such as hexane and toluene, improving their adsorption on the sensor surface.

### 3.3 Effect of Particle Concentration in the Ionogels

**Figure 5:**
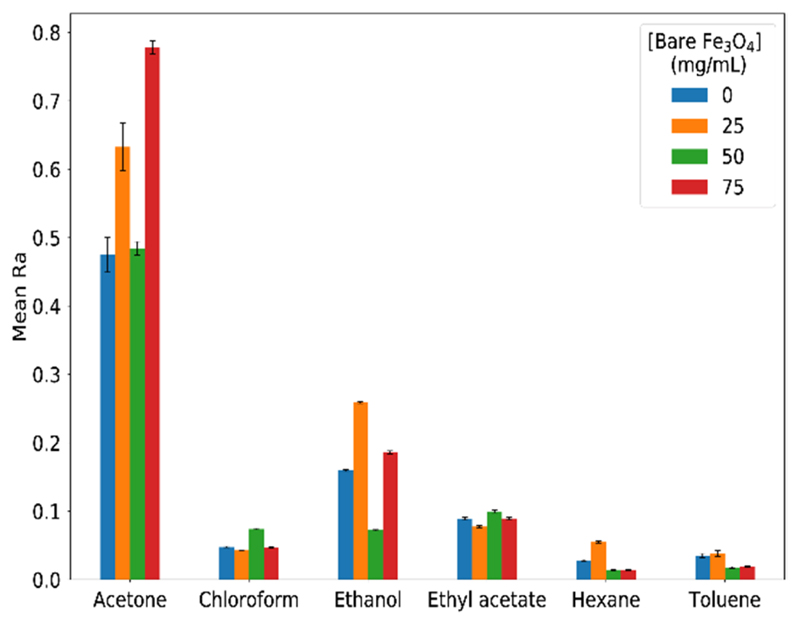
Relative responses of set A sensors upon 10 cycles of exposure to VOCs.

We started by evaluating the amount of particles to be incorporated into the ionogel. Ionogels doped with bare particles in different concentrations (set A sensors) were prepared and used as gas sensors in a custom-made device (Figure 2). The histograms of mean Ra from this set of sensors for the six model VOCs tested are shown in Figure 5. It is possible to observe that the highest mean Ras are those of acetone and ethanol, which are the two most polar VOCs. Acetone has a higher dipole moment (2.88 D) than ethanol (1.69 D) and also a lower boiling point (56 ° C; 78 °C for ethanol). Thir means that at the temperature of the experiment (30 °C), it has a higher vapor pressure and hence more acetone molecules/volume x time reach the sensors, causing higher responses. Looking at the two least responsive VOCs, hexane and toluene, the latter is less volatile but more polar, leading to responses of similar magnitudes. Regarding the concentration of Fe_3_O_4_ particles, the situation is more complex, which generates completely different response patterns for each VOC. It is noteworthy that this is highly desirable, as the ability of the electronic nose, formed by an array of these sensors, to correctly classify the VOCs is based on these response differences.

### 3.4 Effect of Particle Functionalization in Ionogels

As stated above, polar VOCs have higher responses than less polar ones, such as chloroform, ethyl acetate, toluene, and hexane, because of their lower dielectric constants. To increase the response of these VOCs, we functionalized the Fe_3_O_4_ particles with organic compounds that could facilitate the adsorption of these volatiles by the ionogel.

**Figure 6:**
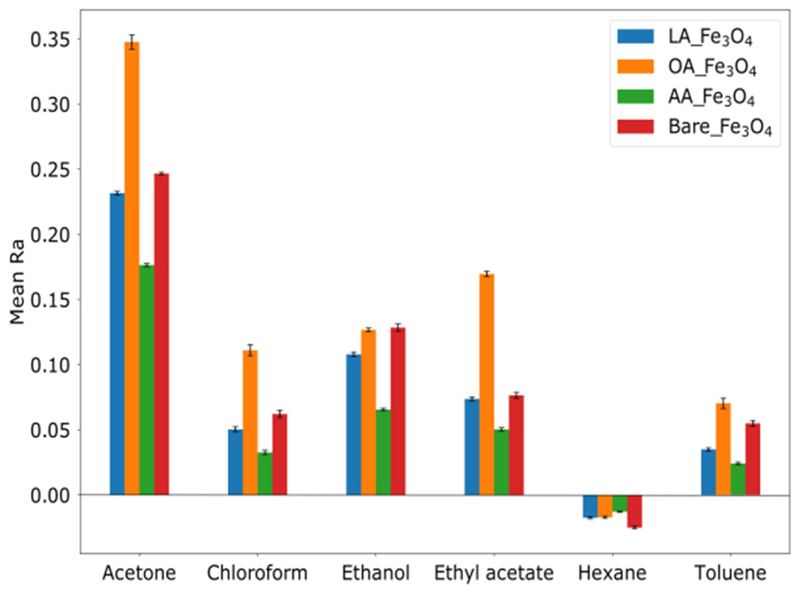
Responses of set B sensors upon 10 cycles of exposure to VOCs.

The concentration of the Fe_3_O_4_ particles was fixed at 25 mg/mL for all the set B ionogels. Figure 6 shows the histograms obtained with the bare and functionalized particles for the tested VOCs. Compared to Figure 5 (bare particles), there is an increase in the response to hexane, toluene, and chloroform, suggesting an improvement in the adsorption to these VOCs, and also a decrease in Ra for the LA ionogel sensor on exposure to acetone and ethanol indicating a decrease in the hydrophilicity of the ionogel. As in set A, each VOC induced a particular response pattern of the sensor set.

### 3.5 Ionogels in e-Nose to Classify Distinct VOCs

**Figure 7:**
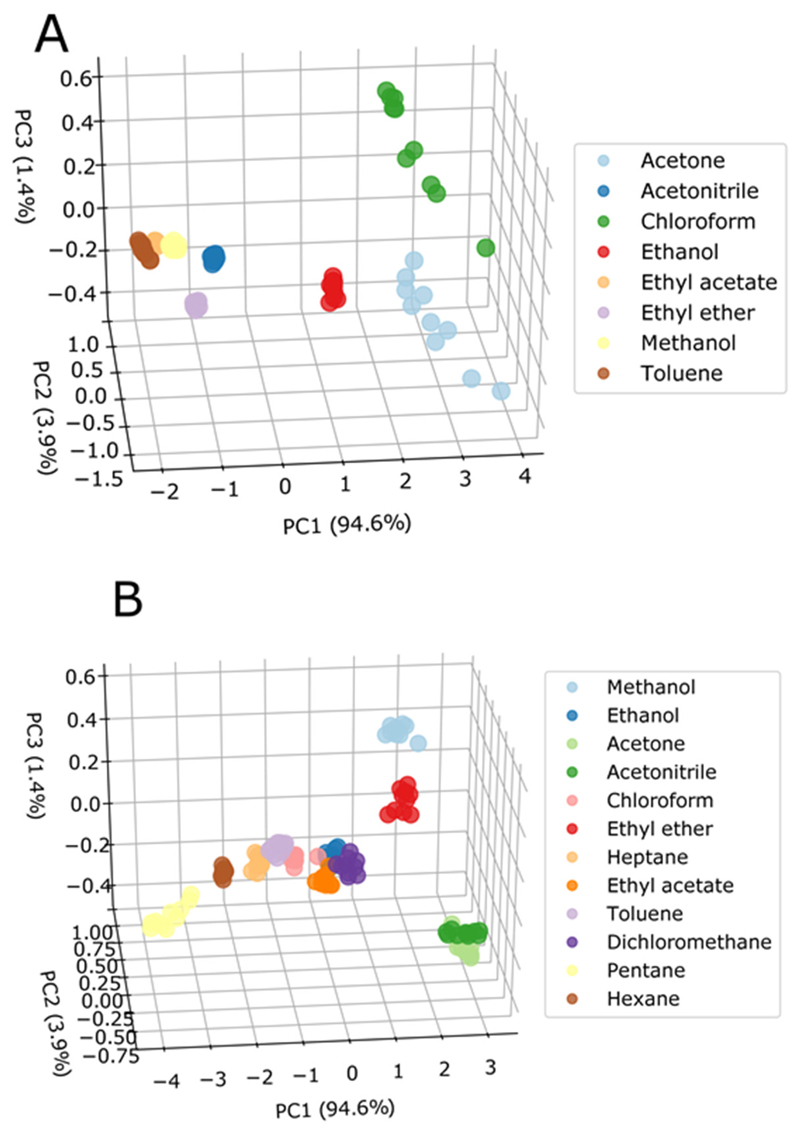
PCA scatter plots indicating the variance of each component (in brackets) for the VOCs tested (PC = principal component); **(A)** with set A sensors and **(B)** with set B sensors.

In order to assess the potential of the new ionogel sensors for the detection and discrimination of VOCs, e-nose experiments were performed with a wider set of model VOCs. Methanol, acetonitrile, dichloromethane, diethyl ether, pentane, and heptane were included in this study. The Ras of the signals obtained for the different VOCs were used as input variables in PCA in order to allow the generation of visualizations in three-dimensional space, since four sensors were used in each set, generating new orthogonal vectors, according with the number of initial dimensions, keeping as much information as possible from the original data [25]. The first three components comprised 99.9% of data coverage for both systems. Figure 6 shows the PCA graphs for both sets of sensors. The sensors composed only of bare particles (set A) were able to distinguish eight out of the 12 VOCs, namely ethyl acetate, acetone, acetonitrile, chloroform, ethanol, methanol, toluene, and diethyl ether (Figure 7A). Although the clusters of some VOCs are close to each other, such as toluene and chloroform, the region of the graph they occupy is quite distinct, without overlapping. The sensors doped with the functionalized particles (set B) were able to distinguish all 12 VOCs, as shown in Figure 7B. VOCs that have structural similarities such as pentane, hexane, and heptane; chloroform and dichloromethane; methanol and ethonol; were grouped in different regions of the graph, indicating the feasibility of the classification by predictive methods.

**Figure 8:**
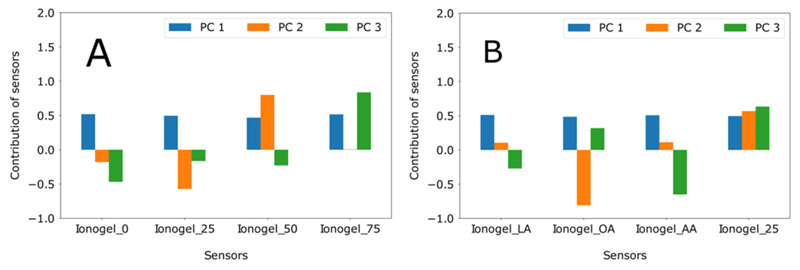
Loading plot showing the contribution of each sensor in the both sets for the principal components, **(A)** with set A and **(B)** with set B.

As mentioned earlier, the PCA generates orthogonal vectors from the input variables. The new coordinates generated by the orthogonal vectors are related to the initial variables by coefficients (loadings), which provide the contribution of each input variable. This means that one can evaluate how strongly a sensor is able to influence the principal components by plotting the loading of each sensor (Figure 8). For sensor set A (Figure 8A), all sensors contribute equally (loading of 0.52, 0.50, 0.47, and 0.52 for ionogel_0, ionogel_25, ionogel_50 and ionogel_75, respectively) to the first component, which has the highest variance. However, for the second component, ionogel_50 and ionogel_25 showed the biggest contribution (loading 0.80 and −0.57, respectively) and for the third component, ionogels 0 and 75 are the most important (loading −0.42 and 0.84, respectively). Analyzing the loading of sensot set B (Figure 8B), it is possible to observe a similar result as of set A for a first component, where all sensors contribute equally (loading 0.51, 0.48, 0.51, and 0.49 for ionogel_LA, ionogel_OA, ionogel_AA, and ionogel_25, respectively). For the second component, ionogels OA and 25 showed major contributions (loading −0.81 and 0.57, respectively) and for the third component, ionogela_AA and 25 are the most important (loadtng −0.65 and 0.63, respectively). It is worth mentioning that in the set of sensors B, the ionogel_25 presented almost the same contribution for the three components, while the other sensors contributed differently for each principal component.

**Figure 9:**
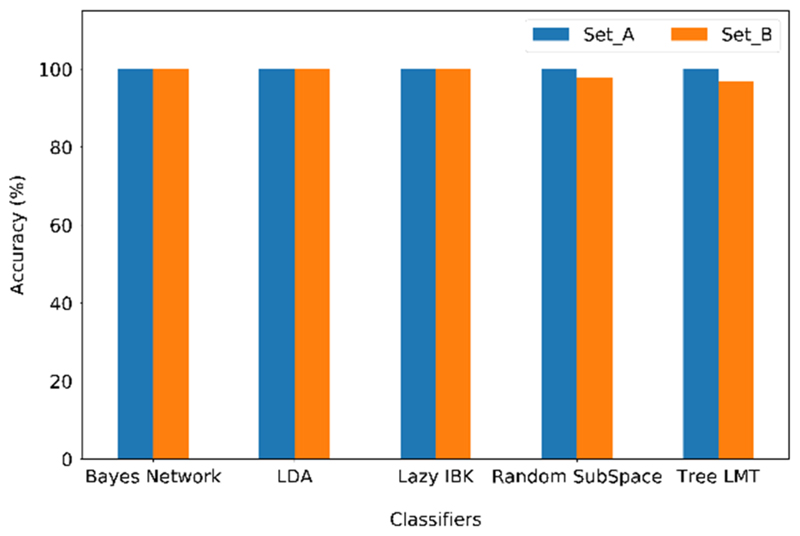
Comparison of the accuracies of the different classifiers used for set A and set B sensors.

Although the separation between the VOC clusters for both sets of sensors is clear in the PCA plots, it is necessary to use a validation method to assess the degree of accuracy in predictions. For this purpose, different classification methods were applied to the dataset, and then the accuracy was calculated for each algorithm and validated by the K-fold cross-validation method. Bayes Network, Linear Discriminant Analysis, Lazy Instance Based (Lazy IBK), Random SubSpace, and Logistic Model Trees (LMT) classifiers were selected using the WEKA software [46]. Set A consisted of 64 measurements in total, with eight repetitions for each VOC, and set B consisted of 96 measurements in total, with eight repetitions for each VOC. Cross-validation used 90% of the data in both sets to train the algorithms and 10% of the data for predictions. The k value of the K-fold method was 10. The correctness percentage for both sets was greater than 98% regardless of the classifier used, as shown in Figure 9. The accuracy of the classifiers for set A was 100% for all tested classifiers, while for set B, there was 100% for Bayes Network, LDA, and IBK classifiers, 97.9% for Random SubSpace, and 96.9% for Tree LMT.

**Figure 10:**
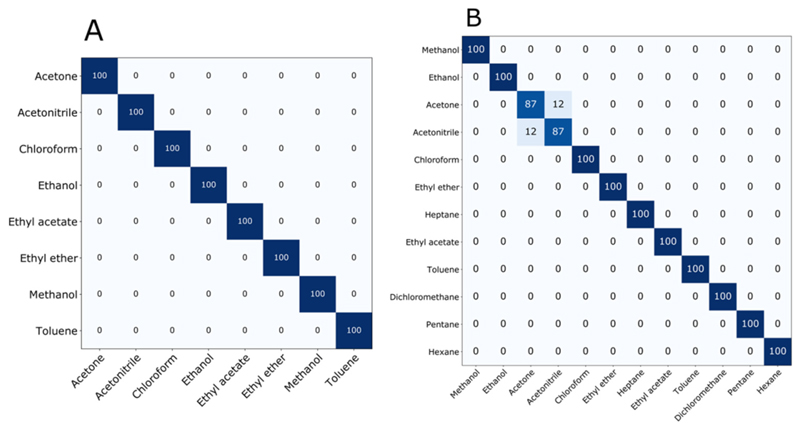
Confusion matrices of the Random SubSpace classifier applied in the predictions of VOCs **(A)** in set A sensors and **(B)** in set B sensors.

Figure 9 shows the accuracy of each classifier, considering the entire dataset. Observing the degree of correctness for each VOC, it is possible to analyze which compounds can be assigned or classified incorrectly. Figure 10 shows the confusion matrix with the percentage of correctness of each VOC for the two sets of sensors studied, using the Random SubSpace classifier. As can be seen in Figure 10A, the hit rate was maximum in the classification of all eight VOCs by set A, while by set B (Figure 10B), it was maximum for 10 VOCs. Acetone and acetonitrile were confused with each other in 12.5% of the tests. Their clusters are fairly close on the PCA plot (Figure 7B), but there is no overlap. Although there is confusion for this classifier, it is important to note that three other classifiers indicated 100% of accuracy, showing that the mistake depends on the selected classifier.

Finally, it is important to mention that many e-noses are based on metal oxide semi-conductors (MOS) or on conductive polymers (CP) or both as their sensing materials [47]. MOS-based sensors are versatile and cost effective but need high temperature to operate (up to 500 °C), which implies relative high power consumption and risk of explosion in contact with certain organic volatiles. On the other hand, CPs have the advantage of room temperature operation, are flexible, and can be adjusted to specific targets. However, their synthesis may be laborious, and they present sensitivity to moisture that has to be compensated. These are important disadvantages of these materials. The sensors based on ionogel composites, used in this study, operate at room temperature and are easy to be produced in only a few steps. Furthermore, the sensors are fast to respond and to recover.

## 4 Conclusions

E-noses were developed from a single ionogel doped with bare or functionalized iron oxide particles. The change in particle concentration produced four sensors that combined in an e-nose were able to detect and distinguish eight VOCs, while ionogels containing a fixed concentration of particles but functionalized with different compounds led to an e-nose capable of differentiating twelve VOCs. This second e-nose showed greater selectivity for less polar VOCs, while sensors with bare particles showed more affinity for polar VOCs. Using different machine learning algorithms and a K-fold cross-validation method, the degree of correctness of the e-noses was evaluated, obtaining accuracies above 98% for the two sets of sensors.

These results show that it is possible to fine-tune the e-nose response to the system that should be analyzed by choosing the compounds that will functionalize the Fe_3_O_4_ particles. This is an advantage since it allows keeping the number of sensors small and hence lowering the overall cost of the equipment. On the other hand, it means that for each type of application, one has to prepare specific sensors, which may be seen as a disadvantage. Probably, an alternative way to enhance the selectivity of the e-nose without increasing the number of sensors may be extracting more features besides the relative response (Ra) such as, for instance, the time that it takes to each sensor to reach half the maximum response, the first derivative of each response curve, etc.

Finally, low cost, ease of production, high selectivity, room temperature operation, low power consumption, and fast response (only 5 s exposure/100 s recovery times) are some of the advantages in the application of this kind of sensing materials. This paper is a proof-of-concept type paper providing preliminary results, since the e-noses were only tested with pure organic solvents as model VOCs. Other types of particle functionalization should be explored in the future to tune the affinity of the sensors for other analytes of interest.

## Figures and Tables

**Table 1:** Preparation of the ionogels.

Entry	Sensor	Fe_3_O_4_ conc. in Water (mg/L)	Fe_3_O_4__coating conc. in Organic Solvent (mg/L)
1	Ionogel_0	-	-
2	Ionogel_25	25	-
3	Ionogel_50	50	-
4	Ionogel_75	75	-
5	Ionogel_AO	0	25
6	Ionogel_AL	0	25
7	Ionogel_AA	0	25

## Data Availability

The data presented in this study are available on request from the corresponding author.

## References

[1] Ghandi K (2014). A Review of Ionic Liquids, Their Limits and Applications. Green Sustain Chem.

[2] Keaveney ST, Haines RS, Harper JB (2017). Ionic liquid solvents: The importance of microscopic interactions in predicting organic reaction outcomes. Pure Appl Chem.

[3] Zolfigol MA, Khazaei A, Moosavi-Zare AR, Zare A (2010). 3-Methyl-1-sulfonic acid imidazolium chloride as a new, Efficient and recyclable catalyst and solvent for the preparation of N-sulfonyl imines at room Temperature. J Iran Chem Soc.

[4] Galiński M, Lewandowski A, Stepniak I (2006). Ionic liquids as electrolytes. Electrochim Acta.

[5] Sasikumar B, Arthanareeswaran G, Ismail AF (2018). Recent progress in ionic liquid membranes for gas separation. J Mol Liq.

[6] Le Bideau J, Viau L, Vioux A (2011). Ionogels, ionic liquid based hybrid materials. Chem Soc Rev.

[7] Andrzejewska E, Marcinkowska A, Zgrzeba A (2017). Ionogels—Materials containing immobilized ionic liquids. Polimery.

[8] Viau L, Tourné-Péteilh C, Devoisselle J-M, Vioux A (2010). Ionogels as drug delivery system: One-step sol-gel synthesis using imidazolium ibuprofenate ionic liquid. Chem Commun.

[9] Zhao K, Song H, Duan X, Wang Z, Liu J, Ba X (2019). Novel Chemical Cross-Linked Ionogel Based on Acrylate Terminated Hyperbranched Polymer with Superior Ionic Conductivity for High Performance Lithium-Ion Batteries. Polymers.

[10] Zhang X, Fu X, Yang S, Zhang Y, Zhang R, Hu S, Bao X, Zhao F, Li X, Liu Q (2019). Design of sepiolite-supported ionogel-embedded composite membranes without proton carrier wastage for wide-temperature-range operation of proton exchange membrane fuel cells. J Mater Chem A.

[11] Zhang J, Zhang W, Guo J, Yuan C, Yan F (2015). Ultrahigh Ionic Liquid Content Supramolecular Ionogels for Quasi-Solid-State Dye Sensitized Solar Cells. Electrochim Acta.

[12] Joshi N, Rawat K, Solanki PR, Bohidar HB (2015). Biocompatible laponite ionogels based non-enzymatic oxalic acid sensor. Sens Bio-Sens Res.

[13] Ersoez B, Bauersfeld M-L, Wöllenstein J (2016). Ionogel—Based Composite Material for CO2 Sensing Deposited on a Chemiresistive Transducer. Proceedings.

[14] Chrobok A, Baj S, Pudlo W, Jarzębski A (2010). Supported ionic liquid phase catalysis for aerobic oxidation of primary alcohols. Appl Catal A.

[15] Vittoz P-F, El Siblani H, Bruma A, Rigaud B, Sauvage X, Fernandez C, Vicente A, Barrier N, Malo S, Levillain J (2018). Insight in the Alginate Pd-Ionogels—Application to the Tsuji-Trost Reaction. ACS Sustain Chem Eng.

[16] Netto MMO, Gonçalves WB, Li RWC, Gruber J (2020). Biopolymer based ionogels as active layers in low-cost gas sensors for electronic noses. Sens Actuators B Chem.

[17] Shi H, Zhang M, Adhikari B (2018). Advances of electronic nose and its application in fresh foods: A review. Crit Rev Food Sci Nutr.

[18] Vos HGJ, Stevan SL, Ayub RA (2019). Peach growth cycle monitoring using an electronic nose. Comput Electron Agric.

[19] Gamboa JCR, Albarracin ES, da Silva AJ, Ferreira TAE (2019). Electronic nose dataset for detection of wine spoilage thresholds. Data Br.

[20] Ezhilan M, Nesakumar N, Babu KJ, Srinandan CS, Rayappan JBB (2019). Freshness Assessment of Broccoli using Electronic Nose. Measurement.

[21] John AT, Murugappan K, Nisbet DR, Tricoli A (2021). An Outlook of Recent Advances in Chemiresistive Sensor-Based Electronic Nose Systems for Food Quality and Environmental Monitoring. Sensors.

[22] Cordeiro JR, Li RWC, Takahashi ES, Rehder GP, Ceccantini G, Gruber J (2016). Wood identification by a portable low-cost polymer-based electronic nose. RSC Adv.

[23] Kumar J, Singh H, Raj VB, Nimal AT, Gupta V, Singh VK (2020). Trace Detection of Nerve Agent Simulant in the Fuel Vapour Environment using Metal Oxide/Surface Acoustic Wave E-Nose. Def Sci J.

[24] Deshmukh S, Bandyopadhyay R, Bhattacharyya N, Pandey RA, Jana A (2015). Application of electronic nose for industrial odors and gaseous emissions measurement and monitoring—An overview. Talanta.

[25] Sun H, Tian F, Liang Z, Sun T, Yu B, Yang SX, He Q, Zhang L, Liu X (2017). Sensor Array Optimization of Electronic Nose for Detection of Bacteria in Wound Infection. IEEE Trans Ind Electron.

[26] Lim SH, Martino R, Anikst V, Xu Z, Mix S, Benjamin R, Schub H, Eiden M, Rhodes PA, Banaei N (2016). Rapid Diagnosis of Tuberculosis from Analysis of Urine Volatile Organic Compounds. ACS Sens.

[27] Santini G, Mores N, Penas A, Capuano R, Mondino C, Trove A, Macagno F, Zini G, Cattani P, Martinelli E (2016). Electronic Nose and Exhaled Breath NMR-based Metabolomics Applications in Airways Disease. Curr Top Med Chem.

[28] Xia QS, Zhang ZC, Liu YJ, Leng JS (2020). Buckypaper and its composites for aeronautic applications. Compos Part B Eng.

[29] Ravishankar B, Nayak SK, Kader MA (2019). Hybrid composites for automotive applications—A review. J Reinf Plast Compos.

[30] Park SJ, Park CS, Yoon H (2017). Chemo-Electrical Gas Sensors Based on Conducting Polymer Hybrids. Polymers.

[31] Zheng YG, Li HY, Shen WF, Jian JW (2019). Wearable electronic nose for human skin odor identification: A preliminary study. Sens Actuators A Phys.

[32] Swe MM, Eamsa-Ard T, Srikhirin T, Kerdcharoen T Monitoring the Freshness Level of Beef Using Nanocomposite Gas Sensors in Electronic nose.

[33] Carvalho T, Vidinha P, Vieira BR, Li RWC, Gruber J (2014). Ion Jelly: A novel sensing material for gas sensors and electronic noses. J Mater Chem C.

[34] Hussain A, Semeano ATS, Palma SICJ, Pina AS, Almeida J, Medrado BF, Pádua ACCS, Carvalho AL, Dionísio M, Li RWC (2017). Tunable Gas Sensing Gels by Cooperative Assembly. Adv Funct Mater.

[35] Semeano ATS, Maffei DF, Palma S, Li RWC, Franco BDGM, Roque ACA, Gruber J (2018). Tilapia Fish microbial spoilage monitored by a single optical gas sensor. Food Control.

[36] Gil-González N, Benito-Lopez F, Castaño E, Morant-Miñana MC (2020). Imidazole-based ionogel as room temperature benzene and formaldehyde sensor. Microchim Acta.

[37] Palma SICJ, Marciello M, Carvalho A, Veintemillas-Verdaguer S, del Puerto Morales M, Roque ACA (2015). Effects of phase transfer ligands on monodisperse iron oxide magnetic nanoparticles. J Colloid Interface Sci.

[38] Da Rocha RT, Gutz IGR, do Lago CL (1997). A Low-Cost and High-Performance Conductivity Meter. J Chem Educ.

[39] Yang K, Peng H, Wen Y, Li N (2010). Re-examination of characteristic FTIR spectrum of secondary layer in bilayer oleic acid-coated Fe_3_O_4_ nanoparticles. Appl Surf Sci.

[40] Moré R, Scholz M, Busse G, Busse L, Paulmann C, Tolkiehn M, Techert S (2012). Hydrogen bond dynamics in crystalline β-9-anthracene carboxylic acid—a combined crystallographic and spectroscopic study. Phys Chem Chem Phys.

[41] Moré R, Busse G, Hallmann J, Paulmann C, Scholz M, Techert S (2010). Photodimerization of Crystalline 9-Anthracenecarboxylic Acid: A Nontopotactic Autocatalytic Transformation. J Phys Chem C.

[42] Vidinha P, Lourenço NMT, Pinheiro C, Brás AR, Carvalho T, Santos-Silva T, Mukhopadhyay A, Romão MJ, Parola J, Dionisio M (2008). Ion jelly: A tailor-made conducting material for smart electrochemical devices. Chem Commun.

[43] Zhu X, Zhang H, Wu J (2014). Chemiresistive ionogel sensor array for the detection and discrimination of volatile organic vapor. Sens Actuators B Chem.

[44] Bonhôte P, Dias A-P, Papageorgiou N, Kalyanasundaram K, Grätzel M (1996). Hydrophobic, Highly Conductive Ambient-Temperature Molten Salts. Inorg Chem.

[45] Seddon KR, Stark A, Torres M-J (2000). Influence of chloride, water, and organic solvents on the physical properties of ionic liquids. Pure Appl Chem.

[46] Hall M, Frank E, Holmes G, Pfahringer B, Reutemann P, Witten IH (2009). The WEKA data mining software. ACM SIGKDD Explor Newsl.

[47] Mirzaei A, Kumar V, Bonyani M, Majhi SM, Bang JH, Kim J-Y, Kim HW, Kim SS, Kim K-H (2020). Conducting Polymer Nanofibers based Sensors for Organic and Inorganic Gaseous Compounds. Asian J Atmos Environ.

